# A Phase-Locking Analysis of Neuronal Firing Rhythms with Transcranial Magneto-Acoustical Stimulation Based on the Hodgkin-Huxley Neuron Model

**DOI:** 10.3389/fncom.2017.00001

**Published:** 2017-01-20

**Authors:** Yi Yuan, Na Pang, Yudong Chen, Yi Wang, Xiaoli Li

**Affiliations:** ^1^Institute of Electrical Engineering, Yanshan UniversityQinhuangdao, China; ^2^School of Control Engineering, Northeastern University at QinhuangdaoQinhuangdao, China; ^3^State Key Laboratory of Cognitive Neuroscience and Learning & IDG/McGovern Institute for Brain Research, Beijing Normal UniversityBeijing, China; ^4^Center for Collaboration and Innovation in Brain and Learning Sciences, Beijing Normal UniversityBeijing, China

**Keywords:** TMAS, phase-locking, Hodgkin-Huxley neuron model, neuronal firing rhythms, neurons

## Abstract

Transcranial magneto-acoustical stimulation (TMAS) uses ultrasonic waves and a static magnetic field to generate electric current in nerve tissues for the purpose of modulating neuronal activities. It has the advantage of high spatial resolution and penetration depth. Neuronal firing rhythms carry and transmit nerve information in neural systems. In this study, we investigated the phase-locking characteristics of neuronal firing rhythms with TMAS based on the Hodgkin-Huxley neuron model. The simulation results indicate that the modulation frequency of ultrasound can affect the phase-locking behaviors. The results of this study may help us to explain the potential firing mechanism of TMAS.

## Introduction

Transcranial magneto-acoustical stimulation (TMAS), a novel brain stimulation technology, can generate an electric current in a static magnetic field by using ultrasonic waves to noninvasively stimulate the neural tissues (Norton, 2003; Yuan et al., 2016b). Compared with transcranial magnetic stimulation, a noninvasive brain stimulation tool that has been used for treating and rehabilitating neurological and psychiatric disorders (Bystritsky et al., 2011; Muller et al., 2012), TMAS has a higher spatial resolution for brain stimulation because the spatial resolution of TMAS is determined by the size of the ultrasonic spot, approximately 2 mm in diameter. In comparison with transcranial focused ultrasound stimulation (tFUS), which has evolved rapidly in recent years, with TMAS one can perform deep stimulation with spatial resolutions of approximately 2 mm (Tufail et al., 2010; Yoo et al., 2011). In our previous study, the *in vivo* animal experimental results showed that TMAS could enhance the effect of tFUS on neuromodulation (Yuan et al., 2016b).

The neuron-the key component of the nervous system-is responsible for transmitting information in the nervous system (Prescott and De Koninck, 2002; Prescott et al., 2008). Neurons receive signals from other neurons, and then these input signals affect the dynamic characteristics of the ion channels on the membrane. If the input signal of the neuron is added to the electromagnetic stimulation, the firing threshold and the firing time of the neuron will be changed, which will result in changes to the neural encoding. Therefore, the study of the relationship between the neuron and external stimulation, on the one hand, will help to reveal and explain the principle of the disruption caused by external stimulation. On the other hand, it can also provide a theoretical basis for effective treatment for the control of neural activity.

Therefore, it is very important to analyze neuronal firing rhythms with TMAS. Here, we investigated the phase-locking characteristics of neuronal firing rhythms with TMAS based on the Hodgkin-Huxley (H-H) neuron model.

## Methods

### Principle of TMAS

In TMAS, when a pulsed ultrasound propagates in an electrolytic fluid (such as soft tissue or tissue fluid), the ions move as a result of the ultrasonic wave. The motion of ions in the presence of a static magnetic field will generate a Lorentz force on the ions. The Lorentz force gives rise to an electric current I_*ext*_ that oscillates at the ultrasonic frequency including fundamental frequency and modulation frequency (Montalibet et al., 2001; Grasland-Mongrain et al., 2013; Ammari et al., 2015).

In our previous study (Yuan et al., 2016b), the relationship between the electric current density J_y_ and ultrasonic intensity, ultrasonic fundamental frequency and magnetostatic fields intensity has been derived, it can be expressed as

(1) $$J_{\text{y}}\approx \sigma B_{x}\sqrt{\frac{2I}{\rho c_{0}}}\sin\left(2\pi ft\right)$$

The value of electric current density J_y_, which corresponds to the electric current I_*ext*_, can be used to stimulate the neuron and was used for simulation in the H-H neuron model. σ is the conductivity of the tissue, and a typical value of the conductivity of tissue is 0.5 Siemens/m (Norton, 2003). *B*_x_, *I*, ρ, c_0_ and f are magnetostatic fields intensity, ultrasonic intensity, tissue density, ultrasound speed, and ultrasonic fundamental frequency.

### H-H neuron model

The electric current, which is generated by ultrasonic waves and a magnetostatic field in an electrolytic fluid and conducts through the tissue fluid to the neuronal membrane, stimulate the neurons as the externally-applied current (I_ext_) in H-H neuron model. The time characteristic of neuronal firing was determined by that of electric current. The H-H neuron model includes the following differential equations (Hodgkin and Huxley, 1952).

(2) $$\begin{array}{l} C_{m}\;\frac{dV}{dt}=I_{ext}-[{\bar{g}}_{Na}m^{3}h(V-V_{Na})+{\bar{g}}_{K}n^{4}(V-V_{K})\\ \;+\;g_{L}(V-V_{L})]\\ \;\frac{dm}{dt}=\varphi [\alpha_{m}(V)(1-m)-\beta_{m}(V)m]\\ \;\frac{dh}{dt}=\varphi [\alpha_{h}(V)(1-h)-\beta_{h}(V)h]\\ \;\frac{dn}{dt}=\varphi [\alpha_{n}(V)(1-n)-\beta_{n}(V)n] \end{array}$$

where Cm is the membrane capacitance, and I_*ext*_ is the externally-applied current generated by the ultrasonic wave and magnetostatic field in nerve tissue. V is the membrane potential and can be expressed as *V* = *V*_*intra*_ − *V*_*extra*_. V_*intra*_ and V_*extra*_ are the intracellular and extracellular potentials, respectively. φ = 3^(*T*−6.3)/10^ modifies the time constants of gating variables depending on temperature T (T = 6.3°C). m and h are the gating variables representing the activation and inactivation of the Na^+^ current, respectively. n is the gating variable representing the activation of the K^+^ current. V_*Na*_, V_*K*_, and V_*L*_ are the equilibrium potentials of the sodium, the potassium and the leak electric currents, respectively. g_*Na*_, g_*K*_, and g_*L*_ are the maximal conductance of the corresponding ionic electric currents. α and β are nonlinear functions of V and given by the following equations:

(3) $$\begin{array}{l} \alpha_{m}(V)=0.1(25-V)/[\exp((25-V)/10)-1]\\ \beta_{m}(V)=4\exp(-V/18)\\ \alpha_{h}(V)=0.07\exp(-V/20)\\ \beta_{h}(V)=1/[\exp((-V+30)/10)+1]\\ \alpha_{n}(V)=0.01(10-V)/[\exp((10-V)/10)-1]\\ \beta_{n}(V)=0.125\exp(-V/80) \end{array}$$

The fixed parameters used for the simulation are listed in Table 1. To make the resting potential equal to zero in the H-H neuron model, the value of the membrane potential was shifted by 65 mV.

**Table 1:** **Fixed parameters for H-H neuron model**.

**Parameters**	**Values**	**Units**
C_m_	1.0	μF/cm^2^
g_Na_	120	mS/cm^2^
g_K_	36	mS/cm^2^
g_L_	0.3	mS/cm^2^
V_Na_	115	mV
V_K_	−12	mV
V_L_	10.59	mV
T	6.3	°C
ρ	1120	kg/m^3^
c_0_	1540	m/s

**Figure 1:**
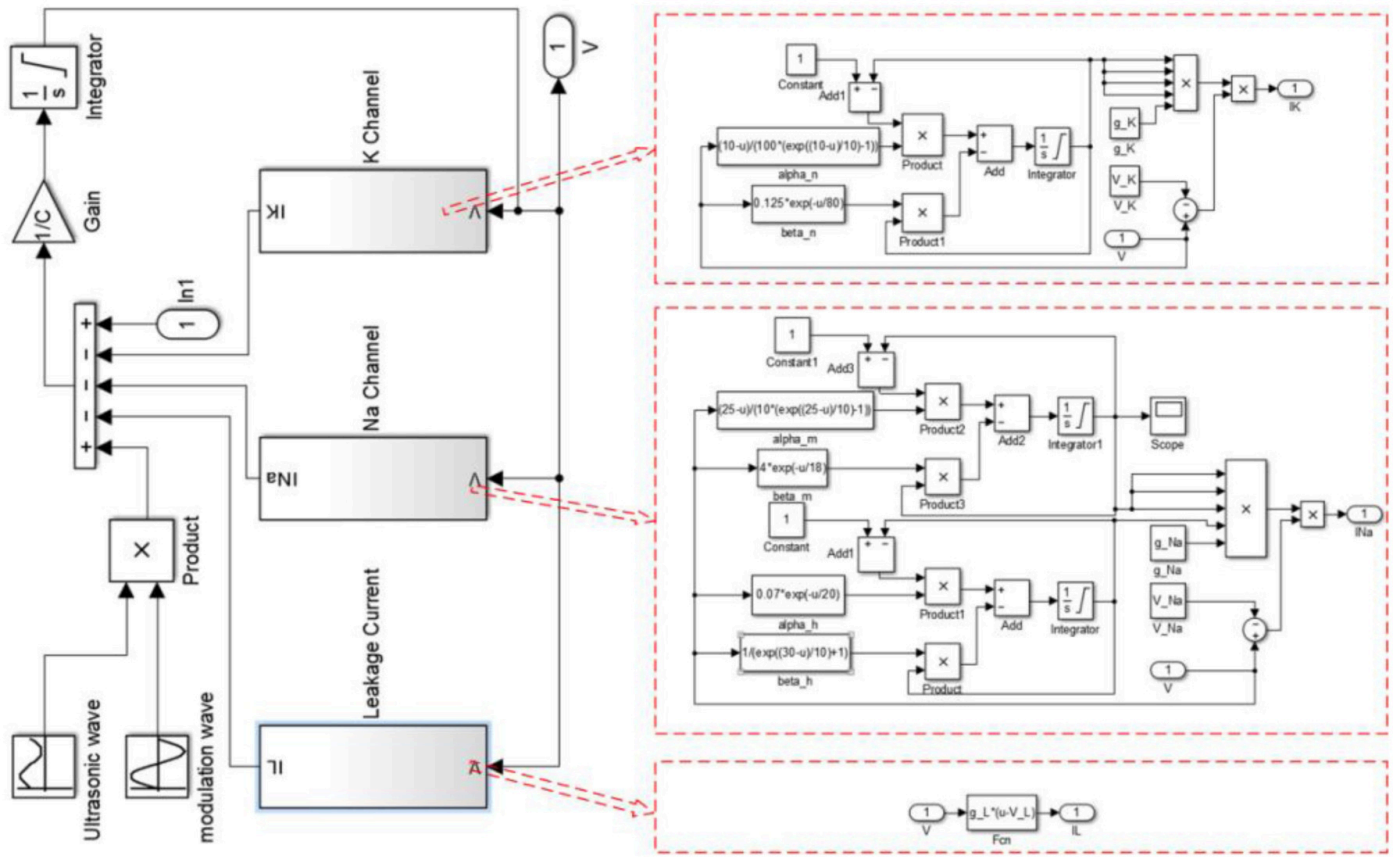
**The structure of Matlab Simulink used for the simulation**.

The simulation was performed with MATLAB Simulink software (2014, MathWorks, USA). The structure of MATLAB Simulink is shown in Figure 1, and the MATLAB code used for the simulation is given in the Supplementary Material.

### Modulation ultrasound

**Figure 2:**
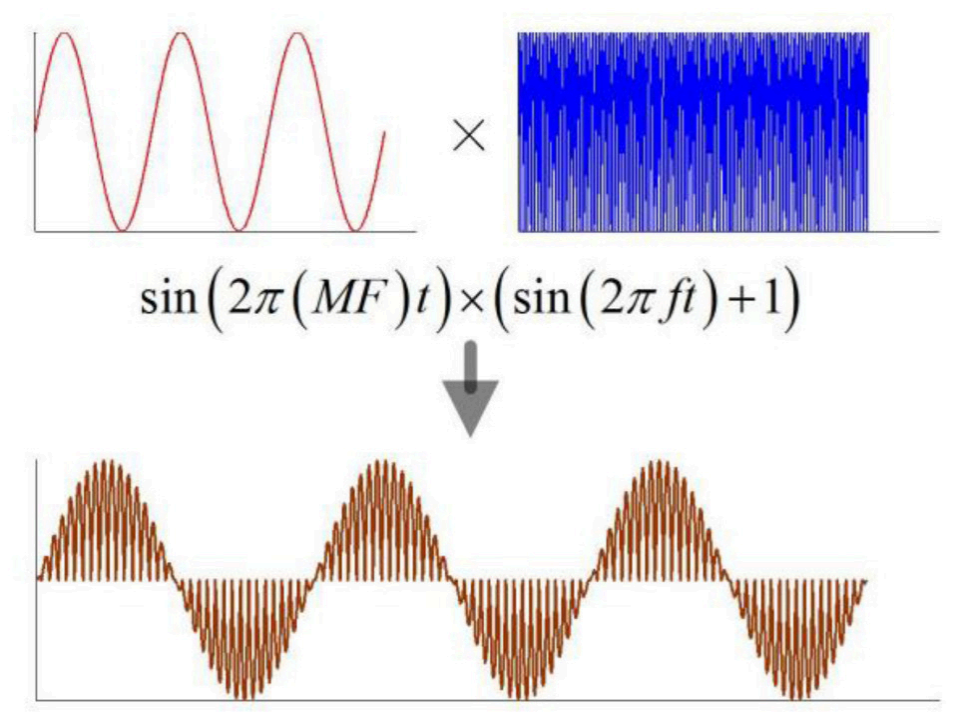
**The schematic of modulation ultrasound, MF, modulation frequency; f, ultrasonic fundamental frequency**.

When the magnetostatic field intensity is constant, the current density in nerve tissue is a function of the ultrasonic intensity according to equation (1). The previous study showed that both continuous and modulated ultrasounds, when used for stimulation of the motor cortex, can safely and repeatedly evoke local field potentials and electromyography activity (Tufail et al., 2011). In this study, a continuous ultrasound with the modulation of a sine wave and a continuous wave were used for simulation (Figure 2). The modulation frequency (MF) ranged from 5 to 150 Hz.

### Average firing rate

The average firing rate of neurons is widely used in the assessment of neuronal information (Carandini and Ferster, 2000; Che et al., 2009). We used the average firing rate to evaluate the encoding information of neuron and identify the relationship between the firing rhythm and the MFs. The average firing rate r of a stimulus cycle is defined as follows:

(4) $$r=\frac{p}{q}$$

where *q* is the number of cycles of the external stimulation, and *p* is the number of neuronal firings in *q* cycles. The average firing rate corresponding to a pattern of *p*:*q* phase-locking is constant and equals *p*:*q*. The *p*:*q* phase-locking, which is a periodic oscillation and defined as *p* action potentials generated by *q* cycle stimulations, is analyzed by calculating the value of *p*:*q*.

## Results

In order to study the effect of TMAS on the neuron, we set the ultrasonic fundamental frequency at 500 kHz and the MFs gradually from 5 to 150 Hz in steps of 0.1 Hz. We keep the ultrasonic intensity at I = 0.15 W/cm^2^ and the magnetostatic field intensity at B = 3 T.

**Figure 3:**
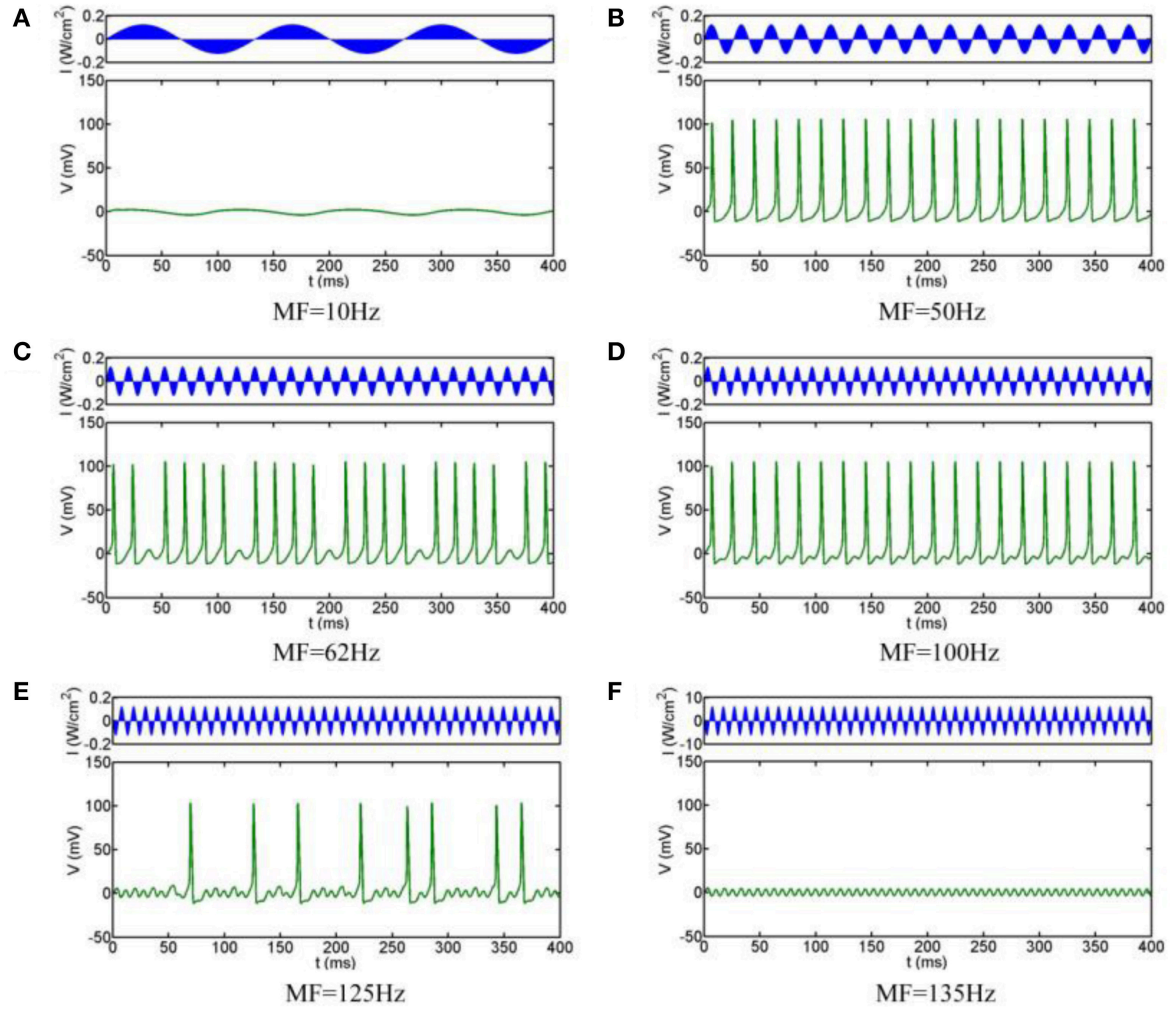
**Firing patterns with different MFs. (A)** 10 Hz, **(B)** 50 Hz, **(C)** 62 Hz, **(D)** 100 Hz, **(E)** 125 Hz, **(F)** 135 Hz.

The simulation results are shown in Figure 3, which clearly shows that the firing pattern of the neurons can be roughly divided into the following typical stages with the increase of the MFs:

- When the MF is small, the neuron does not produce any action potentials, and the oscillation of the transmembrane voltage is small with depolarization under the threshold (e.g., Figure 3A for MF = 10 Hz)
- As the MF gradual increases, a slightly larger frequency can make the neuron excitable: neurons show periodic spiking firing, and the firing frequency remains at the modulation frequency. The state of the system is 1:1 phase-locking, in other words, it is common-frequency phase-locking (e.g., Figure 3B for MF = 50 Hz).
- When the MF is further increased, the neuronal transmembrane voltage is a 23:24 phase-locking oscillation, and then the periodic spiking firing pattern of the neurons is replaced by periodic bursting firing; that is, the 1:1 pattern is replaced by p-1:p (*p* ≥ 2) phase-locking. With the increase of the MF, the phase-locking ratio decreases, as shown in Figure 3C for MF = 62 Hz (phase-locking is equal to 4:5).
- When the MF is larger than 70.7 Hz, phase-locking is equal to 1:2, and the firing pattern changes from the periodic bursting firing pattern to the periodic spiking firing pattern (e.g., Figure 3D for MF = 100 Hz). (v) When the MF increased to 112.2 Hz, the firing pattern is chaotic firing, which means there is no phase-locking. At the mean, the average firing rate varies greatly with the change of timing (e.g., Figure 3E for MF = 125 Hz).
- When the MF is greater than 129.3 Hz, the system return to the state of no action potential (the phase locking is 0:1), the transmembrane voltage oscillates lightly under the threshold, and the fluctuation frequency is consistent with the MF (Figure 3F for MF = 135 Hz).

**Figure 4:**
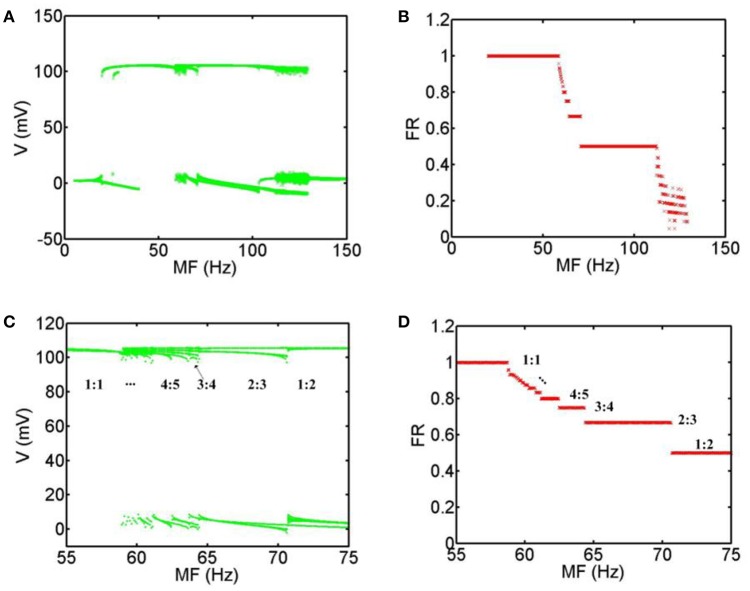
**Bifurcation diagram with MFs. (A)** Bifurcation of the maximum value of the membrane voltage, **(B)** Bifurcation of the average firing rate, **(C)** the magnification of **(A)** in the range of 55–75 Hz, **(D)** the magnification of **(B)** in the range of 55–75 Hz. (MF, modulation frequency; FR, firing rate).

In order to obtain the most accurate relationship between the firing patterns of the neuron and MFs, we calculate the bifurcation diagrams corresponding to the above simulation results. Figure 4A shows that when MF is increased gradually, the general transition procedure of the firing patterns is non-firing (0:1) → spiking firing (1:1) → periodic bursting firing (p-1: p (*p* ≥ 2) phase-locking) → periodic spiking firing (p-1: p (*p* ≥ 2) phase-locking) → chaos → non-firing (0:1). In the range of 5 Hz < MF <19.8 Hz, there are no firing patterns with TMAS. In the range of 19.8 Hz ≤ MF <58.8 Hz, the system exist only with phase-locking oscillations and the average firing rate is equal to 1. When the MF is between 58.8 and 70.7 Hz, the firing pattern of the neurons is periodic bursting firing and the waveform gradually exhibited the bifurcation, and the phase-locking or average firing rate gradually become smaller with the increase of MF. In a certain range of MFs (70.7 Hz ≤ MF <112.2 Hz), the behavior of the system is periodic spiking firing pattern. Figures 4C,D are the magnifications of Figures 4A,B in the range of 55–75 Hz. We can clearly see that the firing patterns form one spike (1:1) and turn into a multiple period spiking (e.g., 4:5, 3:4, 2:3, 1:2). The results are consistent with the previous study with sinusoidal electric field stimulation (Che et al., 2009). In the 112.2 Hz ≤ MF <129.3 Hz regime, the system is in a chaotic state. The distribution of the maximum value of the membrane voltage and of the average firing rate is decentralized and irregular. In the domain of 129.3 Hz < MF ≤ 150 Hz, there are no action potentials with TMAS.

## Discussion

In our study, a continuous ultrasound with the modulation of a sine wave (modulation wave) and a continuous wave (fundamental wave) are used for simulation. There are modulation frequency (5–150 Hz) and fundamental frequency (500 kHz) in the continuous ultrasound. In our previous study, we had demonstrated that the ultrasonic fundamental frequencies from 200 to 700 kHz did not affect the neuronal firing pattern and the neuronal firing rate decreased as the modulation frequency increased from 1 to 100 Hz (Yuan et al., 2016b). Therefore, we only study the effect of modulation frequency on phase-locking of neuronal firing rhythms. In our previous work (Yuan et al., 2016a), we used focused ultrasound with different parameters to stimulate the rat hippocampus. The effect of TMAS on the neuronal firing pattern remains unknown. To address this problem, we investigated the stimulatory mechanism of TMAS on neurons, by using a Hodgkin-Huxley neuron model. The simulation results indicated that the magnetostatic field intensity and ultrasonic intensity affect the amplitude and interspike interval of neuronal action potential under a continuous wave ultrasound. However, we did not pay particular attention to the phase-locking analysis of neuronal firing rhythms with TMAS based on the Hodgkin-Huxley neuron model. Based on the previous study, we focused on phase-locking analysis of neuronal firing rhythms with TMAS and find that TMAS can affect the phase-locking behaviors

We investigate the effect of TMAS on phase-locking analysis of neuronal firing rhythms with different MFs based on the Hodgkin-Huxley neuron model. When the MF is increased, the neuronal firing rhythm are non-firing (5 Hz < MF <19.8 Hz) → spiking firing (19.8 Hz ≤ MF <58.8) → periodic bursting firing (58.8 Hz ≤ MF <70.7 Hz) phase-locking) → periodic spiking firing (70.7 Hz ≤ MF <112.2 Hz) → chaos (112.2 Hz ≤ MF <129.3) → non-firing (129.3 Hz < MF ≤ 150 Hz). These results show that the neuronal firing rhythm is especially sensitive to the change of MF and the increase of MF causes the change of the general transition procedure of the firing patterns.

The neuron exhibits various firing patterns with TMAS under different MFs, for example, periodic spiking firing, periodic bursting firing and chaotic firing. When the neuron exhibits periodic spiking firing and periodic bursting firing, the firing characteristic of the system is phase-locking oscillation. When the neuron exhibits chaotic firing, the neuronal action potential is random, or, put another way, irregular, and the firing characteristic of the system is not phase-locking oscillation. We identified the relationship between the ultrasonic MFs and the neuronal firing pattern and found that the change of the ultrasonic modulation frequency can significantly alter neuronal firing behavior and change the encoding of neural information.

We referenced the research by Che et al. (2009) and used the average firing rate to evaluate the phase-locking analysis of neuronal firing rhythms by TMAS. In future work, we will do the researches using analysis methods (such as time delay embedding and spectral analysis et al) to analyze the effects of TMAS on phase-locking and chaos of neuronal firing rhythms.

There is a large difference of temperature between 6.3°C in H-H neuron model and 37°C of human body. The H-H neuron model provides insight into generic neural behavior, but the results may be different if a model appropriate for a mammalian neuron is used.

The method of TMAS was similar to the electrical stimulation. However, compared with deep brain stimulation that is one of electrical stimulation, TMAS is a noninvasive brain stimulation method. Compared with transcranial direct current stimulation, TMAS has high spatial resolution. In this paper, we only studied the effect of TMAS with sinusoidal ultrasound on neuronal firing rhythm. We will analysis the neuronal firing rhythm induced by TMAS with different ultrasonic types and parameters in the further work.

These results can provide the potential basis for further study of the mechanism of the genesis of the neural firing pattern under different MFs with TMAS.

## Author contributions

YY and XL designed and coordinated the study, YY, NP, YC and YW carried out numerical implementation of the TMAS, YY, NP, YC and YW done the simulation, YY, NP, YC and YW performed data analysis, YY, NP, YC, XL and YW drafted the manuscript, YY, NP, XL and YW edited the manuscript. All authors gave final approval for publication.

### Conflict of interest statement

The authors declare that the research was conducted in the absence of any commercial or financial relationships that could be construed as a potential conflict of interest.
